# Hepatoprotective Effects of Flavonoids Against Acetaminophen Toxicity

**DOI:** 10.3390/molecules31193527

**Published:** 2026-10-03

**Authors:** Arshdeep Panesar, Rukhsana Aslam, Nabila Jahan, Cynthia T. Luk, Apu Chowdhury

**Affiliations:** 1 Keenan Research Centre for Biomedical Science, St. Michael’s Hospital, Unity Health Toronto, Toronto, ON M5B 1W8, Canada; 2 School of Pharmacy, Xi’an Jiaotong University, Xi’an 710061, China

**Keywords:** flavonoids, acetaminophen, hepatotoxicity, antioxidant, anti-inflammation

## Abstract

Acetaminophen (APAP) is a common analgesic and antipyretic agent; however, overdose can cause severe liver injury requiring transplantation or resulting in death. Currently, N-acetylcysteine (NAC) is the standard first-line treatment for APAP-induced liver injury (AILI). There is ongoing interest in exploring complementary hepatoprotective compounds, such as plant-derived flavonoids and their mechanisms of action to improve our understanding and management of AILI. This review aims to identify key flavonoids studied to date in the setting of AILI and their proposed mechanisms. A systematic search was conducted across PubMed, Scopus, Web of Science, and Embase. Eligible studies were restricted to murine models of APAP overdose where isolated flavonoids were administered therapeutically post-APAP-injury. From 1752 identified records, 18 studies investigating sixteen distinct flavonoids met the inclusion criteria. Their major mechanisms of actions were identified which include antioxidation, anti-inflammation, modulation of APAP metabolism, and inhibition of cell death pathways. Furthermore, emerging nano-delivery systems markedly enhanced flavonoid solubility and therapeutic efficacy. Although these findings highlight promising candidates and novel therapeutic targets for AILI, clinical trials are essential to establish human efficacy and safety.

## 1. Introduction

Acetaminophen, also known as paracetamol, N-acetyl para-aminophenol, or APAP, is the most widely used over-the-counter drug for analgesia and antipyresis. In Canada, a total of 3721 cases of APAP overdose were recorded between 2011 and 2019, with 50.3% classified as unintentional poisoning and an overall mortality rate of 1–2% [1,2,3]. Although APAP is considered safe at therapeutic doses, an overdose, whether intentional or accidental, particularly when exceeding 7500 to 10,000 mg per day, can lead to acute liver failure [4]. Individuals with obesity, alcoholic or metabolic dysfunction-associated steatotic liver disease are at higher risk of developing acetaminophen-induced liver injury (AILI) [5].

Following ingestion, acetaminophen is primarily metabolized in the liver; approximately 50% by glucuronosyltransferase, 30% by sulfotransferase, and 5–10% by cytochrome P450 enzymes [6]. The cytochrome P450 enzymes (CYPEs), particularly CYP2E1, convert APAP into N-acetyl-p-benzoquinone imine (NAPQI), a toxic metabolite. NAPQI is normally detoxified through conjugation with glutathione (GSH) and subsequently excreted via bile or plasma. However, in cases of overdose, liver GSH stores become saturated, leading to the accumulation of NAPQI, which binds to cytosolic proteins and causes toxicity [6,7]. Current treatment typically includes the administration of 50 to 100 g of activated charcoal as a single dose for gastrointestinal decontamination, preferably within four hours of ingestion. If serum acetaminophen levels, amount of ingestion or other features suggest a risk of liver injury, N-acetylcysteine should be administered, typically intravenously [8]. Although early administration of N-acetylcysteine (NAC) significantly reduces the risk of severe toxicity, it may cause adverse effects, such as nonallergic anaphylactic reactions, rash, vomiting, bronchospasm, and hypotension [9].

Flavonoids represent a major class of naturally occurring phenolic compounds, with over 3000 known structures. They are abundant in fruits, vegetables, and various plant components, such as bark, leaves, and shoots. Flavonoids have been extensively studied for their antioxidant, anti-inflammatory, anti-mutagenic, anti-cancer, anti-atherosclerosis, anti-asthmatic, anti-neurodegenerative, and hepatoprotective properties, suggesting numerous health benefits [10]. To date, no review has specifically examined the role of flavonoids in the post-injury context of AILI. In this review, “post-injury” refers to studies in which flavonoid administration occurred after exposure to a toxic dose of acetaminophen. This treatment approach closely aligns with clinical scenarios in which patients seek medical care after acetaminophen overdose. However, it is important to note that all studies included in this review have been conducted in animal models. We conducted a systematic search to identify flavonoids with potential therapeutic effects against AILI, covering studies published from 2010 to 2025 (15 years).

This review adheres to strict inclusion criteria, selecting only studies that investigated mice in the setting of AILI treated with whole flavonoids post-injury. This search yielded evidence for specific flavonoids and their underlying mechanisms contributing to their potential therapeutic benefits in AILI.

## 2. Research Methodology

The present study aims to evaluate the use of flavonoids in mouse models of AILI, identify the most promising compounds, and understand their proposed mechanisms of action. The Preferred Reporting Items for Systematic Reviews and Meta-Analysis (PRISMA) revised 2020 guideline was followed [11].

### 2.1. Search Strategy

Controlled searches were conducted in four databases—PubMed, Scopus, Web of Science Core Collection and Embase—to identify potentially relevant publications from 2010 to 2025. All searches were run on 18 September 2026 and were limited to publications in English. The same three concept blocks (flavonoids, acetaminophen and liver injury) were used in every database; because Medical Subject Headings have no equivalent in Scopus or Web of Science, their entry terms were added as free-text terms, and Emtree terms were used in Embase. The full search string for each database is provided in the supplementary data (Supplementary Table S1).

### 2.2. Selection Standards and Data Collection

Two investigators independently screened the eligible publications. Studies were included if they met the following criteria: original research articles using models of AILI and studies involving flavonoid intervention post-injury. The selection process excluded review articles, studies including treatment with whole plant extract without specific identified flavonoids, research lacking an established AILI model, non-English publications, retracted articles, articles with unavailable full text, studies that did not use mice, pre-treatment studies, and studies with unavailable methodology.

A standardized data extraction form was developed to capture the characteristics of studies investigating flavonoids in AILI (Supplementary Table S2).

## 3. Results

**Figure 1:**
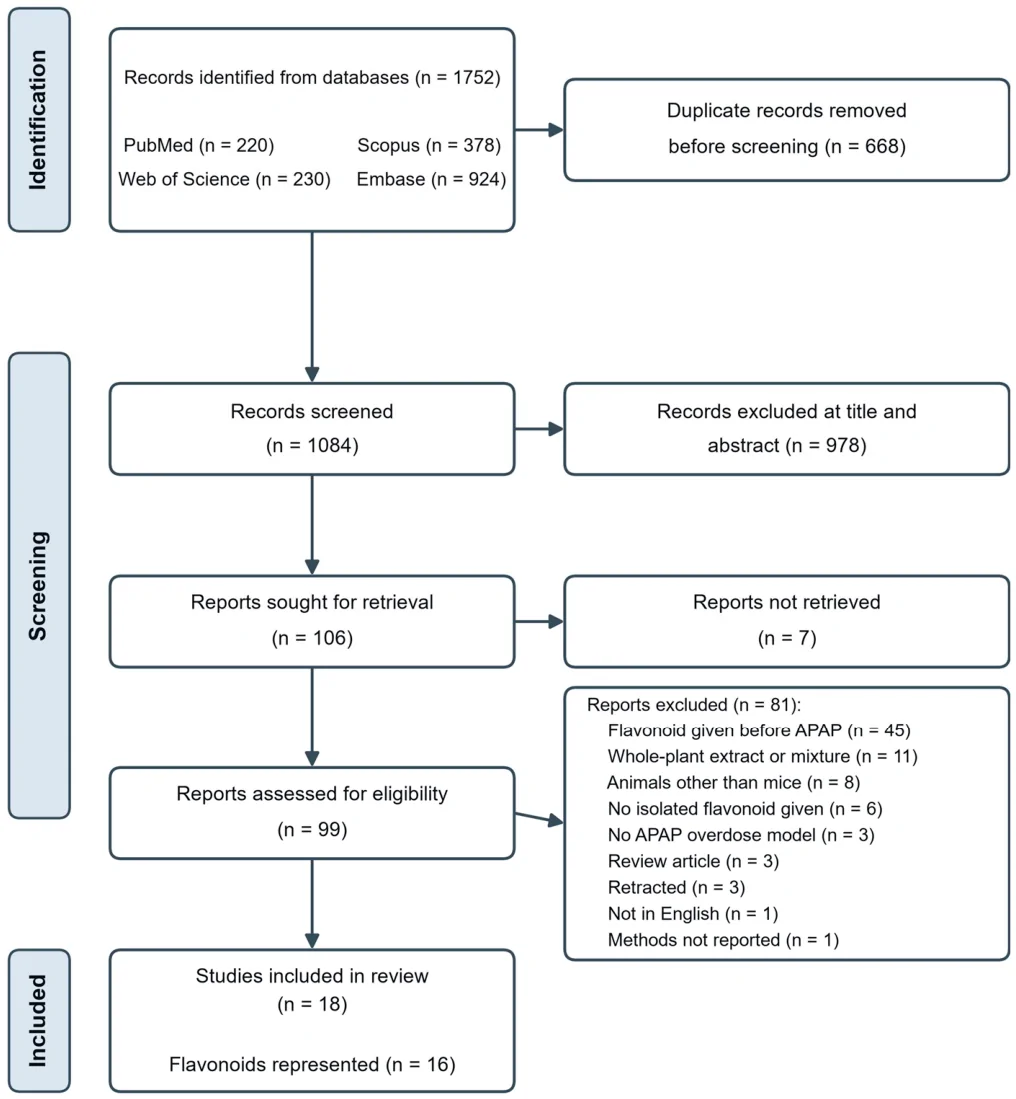
PRISMA 2020 flow diagram illustrating the selection process for studies on flavonoid interventions in acetaminophen-induced liver injury models. A total of 1752 records were identified through searches of PubMed, Scopus, Web of Science and Embase. After duplicate removal, screening and application of the inclusion and exclusion criteria, 18 studies were included in the final review.

A total of 1752 records were identified across the four databases (PubMed, *n* = 220; Scopus, *n* = 378; Web of Science, *n* = 230; Embase, *n* = 924) (Figure 1 and Supplementary Table S3). After 668 duplicates were removed, 1084 unique records were screened. Altogether 978 records were excluded on the basis of their titles and abstracts, as they met the specific exclusion criteria. Common reasons for not including studies were the use of pre-injury rather than post-injury protocols, the use of animals other than mice, the use of whole plant extracts instead of specific flavonoids, or a lack of focus on AILI. Full texts were sought for the remaining 106 reports, of which 7 could not be obtained, leaving 99 reports assessed for eligibility.

After full-text examination of these 99 reports, a further 81 publications were excluded: 45 administered the flavonoid before, or both before and after, acetaminophen (*n* = 45); eleven used total plant or leaf extracts instead of isolated flavonoids (*n* = 11); eight used animals other than mice (*n* = 8); six did not administer an isolated flavonoid (*n* = 6); three lacked an acetaminophen overdose model (*n* = 3); three were review articles (*n* = 3); three were retracted (*n* = 3); one was written in a non-English language (*n* = 1); and one did not provide methodology in the full text (*n* = 1). Pre-injury dosing was therefore the single largest reason for exclusion at full text, accounting for 45 of the 81 excluded reports.

From the final selection of eighteen articles (Table 1), sixteen different flavonoids (genistein, apigenin, silibinin, naringenin, silymarin, baicalin, oroxyloside, narirutin, tiliroside, taxifolin (also known as dihydroquercetin), hesperidin, galangin, epigallocatechin-3-gallate, phloretin, 4-hydroxychalcone and 4′-hydroxychalcone) were identified. Three of these—phloretin, 4-hydroxychalcone and 4′-hydroxychalcone—were evaluated within a single study and act through the same pathways as the remaining compounds.

### 3.1. Antioxidant Effects

During APAP overdose, GSH becomes saturated, leading to excess NAPQI formation, which subsequently forms acetaminophen-cysteine adducts. The overproduction of NAPQI initiates mitochondrial protein adduct formation [12,17,19,20,21,30,31,32,33,34,35,36,37], resulting in oxidative stress and the generation of reactive oxygen species (ROS) and nitric oxide (NO). These reactive molecules activate signaling cascades that drive programmed cell death. Under normal conditions, ROS and NO are attenuated by inherent antioxidant enzymes, including superoxide dismutase (SOD), glutathione peroxidase (GPx), glutathione disulfide (GSSG), manganese superoxide dismutase, and GSH. However, during APAP toxicity, the endogenous antioxidant defense system becomes saturated. NAC, the primary treatment strategy, provides the precursor cysteine to restore GSH and may have other mechanisms of action including binding ROS [38,39].

Flavonoids like genistein, apigenin, silibinin or silibinin-loaded bovine serum albumin nanoparticles (SIL/BSA), naringenin-loaded bovine serum albumin nanoparticles (NGNPs) silymarin, baicalein, and oroxyloside showed potential antioxidant action against APAP intoxication in various studies [12,13,14,16]. Supplementation of genistein, phloretin, 4-hydroxychalcone (4-HC), and 4′-hydroxychalcone (4′-HC) in an AILI animal model decreased serum alanine transaminase (ALT), aspartate transaminase (AST) and increased hepatic malondialdehyde and GSH level [12,29]. Similarly, apigenin, NGNPs, and silymarin treatment helped to attenuate NO and ROS level [13,16,17]. Genistein, silymarin, and baicalin treatments also increased the amount of hepatic and nephrotic antioxidant enzymes GPx, GPx1, Glutathione S-transferase (GST), Glutamate-Cysteine Ligase Catalytic Subunit (GCLC), Glutamate-Cysteine Ligase Modifier Subunit (GCLM), GSSG, and GSH [12,17]. Oroxyloside treatment in female mice after APAP decreased hepatic ROS generation and markers of ER stress [21]. In addition, narirutin, epigallocatechin-3-gallate molybdenum nanoparticles (EGM NPs), and galangin nanoparticles exhibited pronounced antioxidant effects in mice following APAP challenge. Post-injury administration of narirutin significantly elevated the hepatic activities of primary endogenous antioxidant enzymes in mouse liver tissue, including SOD, catalase, and GPX, which were depleted following APAP overdose [22,27,28]. Similarly, the flavanonol taxifolin demonstrated robust post-injury antioxidant rescue when delivered via self-assembling glycymicelles (TAX@pro-glycymicelles) following a toxic APAP challenge. TAX@pro-glycymicelles restored hepatic GSH reserves and SOD activity in mice to baseline control levels, while concurrently suppressing MDA [24].

Similarly, hesperidin formulated as mixed micelles (Hes-MMs) demonstrated pronounced antioxidant defense against APAP-induced hepatotoxicity by effectively attenuating APAP-induced ROS accumulation and preventing the loss of mitochondrial membrane potential. Furthermore, treatment with Hes-MMs restored the expression of key endogenous antioxidant enzymes depleted by APAP overdose, notably manganese superoxide dismutase (MnSOD) and NADPH quinone oxidoreductase 1 (NQO1) [26].

The nuclear factor erythroid 2-related factor 2 (*Nrf2*) is a master antioxidant gene that regulates the transcription of essential genes like *Heme oxygenase-1 (HO-1)*, *GPx2*, *NQO1*, *GCLC*, and *GCLM*. These genes contribute to the downregulation of ROS and xenobiotic detoxification as well as GSH production and regeneration [34]. Upregulation of Nrf2 is a potential approach to treating AILI injury and studies showed that Nrf2 upregulation is a major mechanism for flavonoids. Shi et al. [40] showed that with baicalein treatment, the Nrf2 upregulation occurred by disassociating from kelch-like ECH-associated protein-1 (Keap1), and sustained phosphorylation of protein kinase-C and extracellular protein kinases-1/2 regulation [40]. Cai et al. [23] identified tiliroside, a natural dietary glycosidic flavonoid, as a direct small-molecule Keap1 inhibitor. Mechanistically, tiliroside directly engages the Kelch domain of Keap1, disrupting the Keap1–Nrf2 complex and preventing Keap1-mediated polyubiquitination and proteasomal degradation of Nrf2 without altering baseline *Nrf2* mRNA transcription. This promotes robust Nrf2 nuclear accumulation and triggers the transcription of downstream cytoprotective targets, including *HO-1*, *GCLC*, glutathione peroxidase 4, and ferroptosis suppressor protein 1. Consequently, tiliroside restored hepatic GSH/GSSG ratios and suppressed oxidative markers such as myeloperoxidase, malondialdehyde, and 4-hydroxynonenal adducts in APAP-intoxicated mice [23]. Sirtuin 1 (SIRT 1), is also crucial for Nrf2 nuclear translocation and its induction of antioxidant gene expression. Wang et al. [12] showed that when *Sirt1* was knocked down, Nrf2 nuclear accumulation decreased and worsened markers of AILI, which was improved with the genistein administration [12].

Apigenin can also facilitate the translocation of Nrf2 into the cell nucleus. Zhang et al. [13] showed that Apigenin significantly increased the nuclear translocation of Nrf2. This suggests that apigenin prevents acetaminophen-induced hepatotoxicity by increasing Nrf2 expression, decreasing oxidative stress and promoting cellular repair mechanisms [13]. The same study placed this effect downstream of AMPK/GSK-3β signalling and carnitine palmitoyltransferase 1A (CPT1A) activity, as the selective AMPK inhibitor compound C abolished the protection [16]. In the same way, dihydroquercetin (also known as taxifolin), demonstrated potent antioxidant properties in AILI through significantly upregulated the hepatic mRNA expression of *Nrf2* and mitochondrial superoxide dismutase 2 [25].

Cellular oxidative stress can facilitate caspase-9 activation [41]. Caspase-9, which is known for its role in apoptosis, can also remodel mitochondria and increase ROS production by cleaving BH3-interacting domain death agonist (BID) into truncated BID, while caspase-3 inhibits ROS production [41]. After SIL/BSA nanoparticle treatment, intracellular glutathione reductase and antioxidant enzyme activities were greatly enhanced, and SOD reactivity was weakened compared to the APAP group. Additionally, SIL/BSA nanoparticles upregulated Nrf2/ARE antioxidative pathways, significantly increasing the expression of Nrf2, GCLM, HO-1, and NQO1, which were crucial in protecting cells from oxidative stress and damage [14]. These studies identify mechanisms whereby flavonoids attenuate oxidative stress in models of AILI.

### 3.2. Inflammation

In the context of AILI, hepatic Kupffer Cells become activated and release IL-1β, IL-6, and TNF-α. Specifically, IL-1β and IL-6 stimulate hepatocytes to activate immune functions. IL-6 plays a crucial role in the tissue damage response and enhanced IL-1β secretion. TNF-α aids in liver regeneration by triggering IL-6 and initiating apoptosis [37]. In toxin induced liver injury, T lymphocytes including γδT cell infiltrate into injured tissue and secrete IL-17 to recruit neutrophils [42].

In addition to classical cytokine suppression, recent work indicates that flavonoids can mitigate AILI by modulating sterile inflammatory DNA-sensing machinery. Dong et al. [26] demonstrated that Hes-MMs suppressed sterile hepatic inflammation by intercepting the cytosolic mitochondrial DNA (mtDNA)–cGAS–STING axis. Following APAP-induced hepatocellular stress, damaged mitochondria release mtDNA into the cytosol, where it functions as a damage-associated molecular pattern (DAMP) to activate the cytosolic sensor cyclic GMP-AMP synthase (cGAS) and its adaptor stimulator of interferon genes (STING). Administration of Hes-MMs markedly inhibited cytosolic mtDNA leakage and downregulated cGAS and STING protein levels, resulting in significant reductions in pro-inflammatory cytokines, including IL-1β, IL-6, TNF-α, and IFN-γ in the liver [26].

Baicalin plays a role in the inflammatory response following acetaminophen-induced liver injury by enhancing Nrf2 accumulation in the cytoplasm, which facilitates the activation of the NLRP3 inflammasome. This activation leads to increased hepatic expression of IL-18, which subsequently promotes hepatocyte proliferation and liver regeneration. Shi et al. [20] demonstrated that baicalin administration in APAP-intoxicated mice increased hepatic IL-18 levels and enhanced the proliferation of hepatocytes, a process that was diminished upon inhibition of NLRP3 inflammasome activation [20]. Baicalin treatment also decreased IL-17 and its production by γδT cells recruited in AILI settings [19]. Other studies showed that administration of apigenin and dihydroquercetin effectively reversed the elevations of IL-6 and TNF-α both in the serum and liver of mice, which are typically elevated during AILI [13,25]. Similarly, tiliroside administration also alleviated APAP-induced hepatic macrophage accumulation and hepatic mRNA transcription of key pro-inflammatory mediators, including *MCP-1*, *IL-1*, *IL-6*, and *TNF-α* [23]. Yang et al. [28] showed that EGM NPs significantly reduced hepatic macrophage infiltration and suppressed the hepatic production of pro-inflammatory cytokines (TNF-α and IL-6) and downregulated the activity of inducible nitric oxide synthase and myeloperoxidase, thereby intercepting the inflammatory feed-forward loop that exacerbates APAP-induced tissue damage [28].

Hepatocellular necrosis following APAP overdose prompts the extracellular release of damage-associated molecular patterns that amplify downstream sterile inflammation. Recent work by Dong et al. [24] demonstrated that post-injury oral treatment with taxifolin-loaded glycymicelles (TAX@pro-glycymicelles) suppressed APAP-induced sterile inflammatory cascades by intercepting the high-mobility group box 1 (HMGB1) signaling axis in murine livers. Elevated hepatic levels of extracellular HMGB1 were markedly reduced by TAX@pro-glycymicelles in a dose-dependent manner, culminating in the significant downregulation of pro-inflammatory cytokines, including TNF-α and IL-6, with IL-6 suppression exceeding the therapeutic efficacy of standard N-acetylcysteine [24].

### 3.3. Metabolic Pathways

CYPEs play a critical role in the metabolism of APAP, facilitating the biotransformation of APAP into the highly reactive and toxic metabolite NAPQI, which contributes to AILI. Multiple CYPE isoforms, including CYP2E1, CYP1A2, CYP2A6, CYP3A4, and CYP2D6, are involved in APAP metabolism, each exhibiting distinct enzymatic activities that differentially influence hepatotoxicity [36].

Silymarin treatment, with or without the combination of other non-flavonoids, has been shown to result in improved serum ALT, AST, blood urea nitrogen, creatinine, tissue NO, SOD, GSSG weight loss, and mortality burden in models of AILI [17].

AILI is associated with reduced hepatic blood flow and increased liver volume. This worsens liver injury by impairing liver function and promoting tissue damage [43]. However, treatment with NGNPs has been shown to reverse these changes. Specifically, in an AILI animal model, NGNPs effectively reduced liver volume and restored rate of hepatic blood flow to normal levels, thereby mitigating the severity of liver injury and enhancing overall liver health [16].

### 3.4. Cell Death Pathways

Apoptosis, programmed cell death, plays a complex role in AILI, distinctly influencing the outcomes of toxicity scenarios. In AILI mouse models, there are increased levels of hepatic caspases-3 and -9, which are key components of the apoptotic pathway [44]. Caspase-3 is a crucial protease and the final executor of apoptotic death in mammalian cells, capable of directly cleaving a wide range of vital structural and functional proteins. Caspase-9, on the other hand, activates Caspase-3 [45]. SIL/BSA nanoparticles decreased apoptosis by inhibiting the activities of both caspase-3 and caspase-9 [14]. This inhibition was associated with decreased apoptotic death in liver cells and reduced liver injury. Similarly, female mice with AILI treated with oroxyloside had lower hepatic TUNEL and pro-apoptotic proteins like cleaved caspase 3, caspase 9, as well as high levels of anti-apoptototic B-cell leukemia/lymphoma 2 protein (Bcl-2) [21].

Dihydroquercetin, NGNPs, and EGM NPs significantly modified the expression of apoptotic markers in the liver. Specifically, reduced the levels of the pro-apoptotic protein Bcl-2-associated X protein while increasing the levels of the anti-apoptotic protein Bcl-2. This decreased the number of TUNEL-positive cells in the liver identified after acetaminophen administration [16,25,28].

SIL/BSA nanoparticles reduced APAP-induced cell death by decreasing ROS accumulation, maintaining mitochondrial homeostasis, and inhibiting the caspase-9/3 cascade pathway [14].

Beyond direct caspase cascade modulation, enhancing mitochondrial quality control through selective autophagy (mitophagy) represents an essential protective mechanism against hepatocyte death in AILI. Treatment with Hes-MMs significantly upregulated the expression of key mitophagy regulators, including PINK1, Parkin, and p62. By promoting the autophagic degradation and clearance of permeabilized, dysfunctional mitochondria before catastrophic membrane rupture, hesperidin prevented excessive cell death and tissue necrosis in APAP-challenged murine livers [26]. Similarly, enhancing organelle-specific clearance via the autophagy-lysosomal pathways and mitophagy prevents apoptotic and necrotic downstream cascades. APAP intoxication leads to sustained MAPK/JNK phosphorylation and mitochondrial translocation, inducing mitochondrial outer membrane permeabilization and the release of cytochrome c (CYCS) into the cytosol to execute cell death. Narirutin significantly attenuated JNK activation and blocked cytosolic CYCS release post-injury [22].

Narirutin acts through a related but distinct arm of autophagy. Fang et al. [22] showed that narirutin, given one hour after acetaminophen, activates transcription factor EB (TFEB) by targeting PPP3/calcineurin, driving TFEB nuclear translocation and the transcription of genes governing autophagy, lysosomal hydrolases, lysosomal membrane proteins and lysosomal acidification. This was accompanied by accelerated clearance of acetaminophen–cysteine protein adducts and reduced MAPK/JNK activation, and the protective effect was abolished in hepatocyte-specific *Tfeb* knockout mice [22].

APAP hepatotoxicity engages complex programmed cell death networks beyond classical caspase signaling, involving overlapping signatures of apoptosis, necroptosis, and lipid peroxidation-driven ferroptosis. In APAP-challenged mice, tiliroside reversed aberrant gene expression associated with ferroptotic and necrotic cell death, significantly downregulating pro-oxidant drivers such as acyl-CoA synthetase long-chain family member 4 [23].

## 4. Discussion

**Figure 2:**
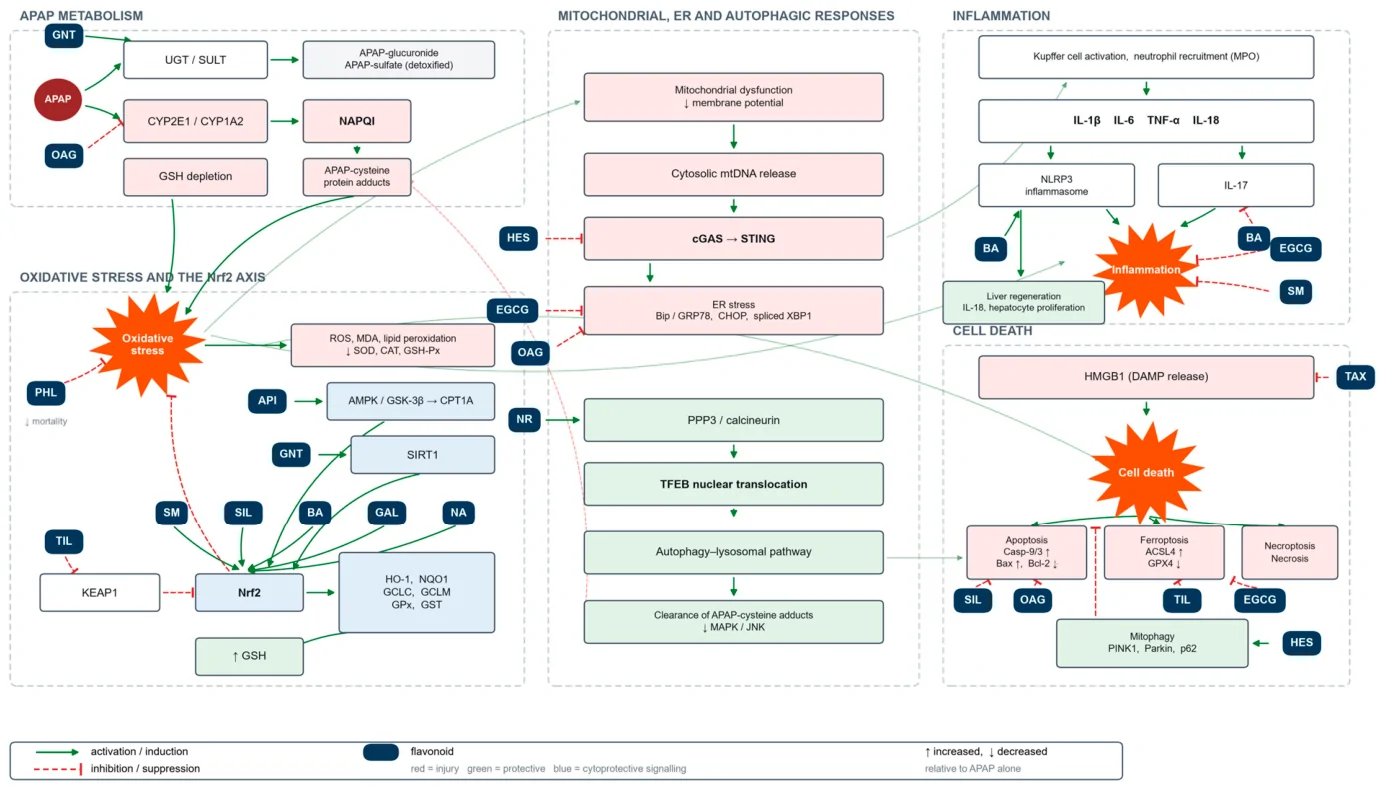
Proposed mechanisms of flavonoid action in acetaminophen-induced liver injury (AILI). During acetaminophen (APAP) overdose, hepatic cytochrome P450 enzymes (CYP2E1, CYP1A2) convert APAP into the reactive metabolite N-acetyl-p-benzoquinone imine (NAPQI), which depletes glutathione (GSH), forms APAP-cysteine protein adducts and drives oxidative stress, mitochondrial dysfunction, inflammation and cell death. Green arrows denote activation or induction; red bars denote inhibition or suppression. Oroxyloside (OAG) limits CYP-mediated bioactivation, while genistein (GNT) induces UDP-glucuronosyltransferase-mediated glucuronidation, an effect dependent on SIRT1. Silymarin (SM), silibinin (SIL), baicalin (BA), galangin (GAL) and naringenin (NA) activate Nrf2 and raise GSH, naringenin acting through the Nrf2/HO-1 axis; tiliroside (TIL) does so by inhibiting KEAP1. Apigenin (API) acts upstream, through AMPK/GSK-3β and CPT1A, with Nrf2 downstream. Hesperidin (HES) blocks cytosolic mitochondrial DNA sensing by cGAS–STING and promotes PINK1/Parkin-dependent mitophagy. Epigallocatechin-3-gallate (EGCG) scavenges reactive oxygen species, relieves endoplasmic reticulum stress and limits apoptosis. Narirutin (NR) activates transcription factor EB (TFEB) via PPP3/calcineurin, driving autophagy–lysosomal clearance of APAP-cysteine adducts. Taxifolin, also known as dihydroquercetin (TAX), intercepts HMGB1-driven sterile inflammation. BA suppresses IL-17-mediated inflammatory injury, yet selectively activates the NLRP3 inflammasome to induce IL-18 secretion, thereby promoting hepatocyte proliferation and hepatic regeneration; TIL and EGCG limit ferroptotic and apoptotic cell death. OAG also relieves endoplasmic reticulum stress. Phloretin (PHL), studied alongside 4-hydroxychalcone and 4′-hydroxychalcone, reduces overall toxicity and mortality. Collectively these flavonoids act on APAP metabolism, oxidative stress, inflammation, autophagy and cell death.

Sixteen flavonoids were identified from eighteen studies as having hepatoprotective properties in models of AILI (Table 1): genistein, apigenin, silibinin, naringenin, silymarin, baicalin, oroxyloside, narirutin, tiliroside, taxifolin (also known as dihydroquercetin), hesperidin, galangin, epigallocatechin-3-gallate, phloretin, 4-hydroxychalcone and 4′-hydroxychalcone. These compounds exhibit their protective effects through diverse mechanisms, including antioxidant, anti-inflammatory, metabolic, anti-apoptotic, autophagic and innate immune signalling pathways (Figure 2). Notably, some flavonoids act through multiple pathways and have been shown to modulate serum levels of alanine transaminase (ALT) and aspartate aminotransferase (AST), key markers of liver injury.

### 4.1. Other Potential Clinical Applications of Flavonoids

This review complements our understanding of the potential roles of these flavonoids in other clinical contexts. Beyond AILI, these flavonoids have been investigated for their therapeutic potential in various diseases. Genistein has been studied for its potentially useful effects in hormone-responsive breast cancer, insulin resistance and cardiovascular risk [46,47,48]. Apigenin has shown promising neuroprotective and nephroprotective effects [49,50,51]. Silibinin has been evaluated for reducing recurrent Hepatitis C virus in patients [52]. Silymarin has been studied for the treatment of metabolic dysfunction-associated steatotic liver disease (MASLD) and may potentially improve cardiovascular risk factors [53,54]. Naringenin has been explored for its benefits in cardiovascular health, endothelial function, and weight management [55,56,57]. Baicalein has been studied for antiviral effects and has also exhibited neuroprotective, antimicrobial, and anti-inflammatory properties [58,59,60,61]. These studies highlight the potential of flavonoids in translational research extending from work in models of acute liver injury.

### 4.2. Flavonoid Delivery Challenges and Advanced Formulation

While flavonoids may have many potential applications, improving their delivery in humans remains a challenge. An interesting aspect of flavonoids is their multiple binding sites and high affinity to the target sites, which may promote efficacy. However, high concentration required to achieve beneficial effects can limit their use. For example, Cao et al. [62] reported that luteolin (polyphenolic flavone) and quercetin (flavanol) are not feasible for human use due to their high IC_50_ values, which exceed human tolerability for consumption as a dietary supplement [62].

One approach to overcome this problem may be nanoparticle-mediated drug delivery systems, enabling a specific molecule to be delivered more precisely. For instance, Hes-MMs, formulated by encapsulating hesperidin into mixed micelles composed of polyvinylpyrrolidone (PVP K17) and Poloxamer 188 improved its aqueous solubility by over 100-fold and achieved near 100% encapsulation efficiency. This nano-delivery system significantly enhanced intestinal permeability and organelle-specific cellular uptake, conferring both prophylactic and therapeutic efficacy against APAP-induced acute liver injury [26].

Tzankova et al. [63] showed that when quercetin was encapsulated in chitosan/alginate nanoparticles in AILI rat model, its stability and bioavailability increased resulting in improved AILI markers [63]. Similarly, SIL/BSA, NGNPs, Fisetin (flavanol) in dipotassium glycyrrhizinate as a nanocarrier [64], silymarin nanoprecipitation in polyvinyl alcohol stabilized Eudragit RS100^®^ polymer [65], silibinin, naringenin and mesoporous silica nanoparticles [66] showed enhanced therapeutic efficacy. Moreover, Gheybi et al. [15] engineered PEGylated silibinin nanoliposomes (SLB-Lip) complexed with soybean phosphatidylcholine, achieving approximately 40% entrapment efficiency and sustained release over 150 h with negligible cytotoxicity [15].

Another strategy to improve oral bioavailability is the lecithin-based carrier system. When silymarin was incorporated with the phytosomal–liposomal carrier system, it improved solubility and bioavailability, specifically targeting the liver for hepatoprotection from APAP exposure [67]. Self-nano emulsifying drug delivery systems (SNEDDS) are natural or synthetic oils, surfactants, and cosurfactants that spontaneously emulsify in the aqueous gastrointestinal tract medium to form an oil in water emulsion with nanosized globules. SNEDDS improves drug solubility, minimizes drug-food interactions, and increases drug bioavailability. Quercetin was optimized with SNEDDS and showed protection against AILI [68].

Natural amphiphilic phytochemicals have also garnered considerable attention as bioactive nanocarriers that eliminate the requirement for synthetic polymers or hazardous organic solvents. For instance, dipotassium glycyrrhizinate (DG), an amphiphilic salt of glycyrrhizin on the FDA Generally Recognized as Safe (GRAS) list, was utilized to formulate TAX@pro-glycymicelles via ethanol evaporation. Encapsulation increased the apparent aqueous solubility of taxifolin by over 40-fold and showed prominent therapeutic effects against AILI [24].

Transition-metal coordination and nanozyme engineering represent another novel avenue for enhancing flavonoid bioactivity in acute hepatic disorders. Yang et al. [28] synthesized ultra-small EGCG-molybdenum nanoparticles via a green one-step redox reaction wherein EGCG served simultaneously as the reducing agent and surface-capping ligand. This demonstrated exceptional organ biocompatibility and targeted liver protection following delayed post-injury administration [28]. Thus, a variety of strategies to improve the delivery of flavonoids are under investigation.

### 4.3. AILI Preclinical Study Caveats

Limitations to existing research on flavonoids in models of AILI include the use primarily of animal models; further studies are required to fully understand the effect of these compounds on AILI in humans. Except for one study that used only female mice [21], most studies used male mice, because male mice are more susceptible to AILI [7], thus it is unclear if flavonoids may have sex-specific effects. Work is needed to better understand the pharmacokinetics of these compounds and ensure their safe and targeted delivery to the liver. Various plant extracts contain flavonoids and non-flavonoid compounds that were studied under AILI conditions with beneficial effects, but the primary active compound isolation and its sole impact were not investigated [69,70].

Several caveats should be considered when interpreting the preclinical evidence for flavonoids in APAP-induced liver injury. Substantial methodological heterogeneity exists across published protocols, particularly regarding the toxic APAP challenge dose, the route of administration, the therapeutic window between intoxication and treatment, and the terminal endpoint timing. Consequently, surrogate biomarkers of hepatic injury, such as serum aminotransferases, cannot be directly compared across different experimental models. Furthermore, most flavonoids are currently supported by solitary investigations, with robust genetic loss-of-function validation established for only a few molecules specifically, hepatocyte-specific *Nrf2* knockout for tiliroside, *Tfeb* knockout for narirutin, and *Sirt1* knockdown for genistein. For many other compounds, reported pathways remain correlative rather than definitively demonstrated through targeted pathway disruption. In addition, several recent evaluations utilized advanced delivery platforms including mixed micelles, liposomes, and inorganic nanoparticles meaning the observed therapeutic efficacy may represent the combined pharmacokinetics and intrinsic hepatoprotective properties of the carrier matrix rather than the unformulated flavonoid alone.

## 5. Conclusions

In conclusion, this review identifies preclinical evidence for sixteen flavonoids that confer significant hepatoprotection when administered post-injury in mouse models of acetaminophen overdose. Rather than acting solely as antioxidants, these compounds exert multiple therapeutic effects. They accelerate the clearance of toxic protein adducts and damaged organelles via autophagy and mitophagy, drive Nrf2-mediated antioxidant transcription, intercept sterile inflammation via HMGB1 and mtDNA–cGAS–STING blockade, and suppress programmed cell death networks, including apoptosis, necroptosis, and ferroptosis. Furthermore, emerging nano-formulation platforms have demonstrated substantial efficacy in overcoming natural bioavailability and solubility barriers. While these findings offer valuable mechanistic insights and highlight novel therapeutic candidates, systematic pharmacokinetic validation and clinical trials remain imperative to translate these preclinical benefits into human therapy.

## Figures and Tables

**Table 1:** Flavonoids identified, with dosing regimen and timing relative to acetaminophen, study duration, the change in serum ALT and AST relative to the APAP-only group, and principal biochemical outcomes. PI (post-injury); values marked ≈ were estimated from the published figures.

Name	Classification	Treatment (Dose, Administration Time After PI)	Treatment Duration	Liver Enzyme vs. Control	Major Outcome	Ref.
Genistein	Isoflavone	Genistein (75–150 mg/kg), 2 h	3–24 h	ALT ≈ −85%, AST ≈ −86%	↑ Sirt1, Nrf2, GSH ↓ MDA	[12]
Apigenin	Flavone	Apigenin (20 or 80 mg/kg, p.o.), 4 h	8 h	ALT ≈ −49%, AST ≈ −49%	↓ MDA, MPO, ROS, IL-6, TNF-α ↑ AMPK/GSK-3β, CPT1A, Nrf2	[13]
Silibinin	Polyphenolic flavonoid	SIL/BSA nanoparticles (20 mg/kg, i.v.), 30 min silibinin nanoliposomes (15 mg/kg, i.v. or i.p.), 2 and 8 h	12 h; 24 h	ALT ≈ −76%, AST ≈ −74%; nanoliposomes ≈ −15%	↓ ROS, MPO^+^ neutrophils, cleaved caspase-9/3, necrosis ↑ Nrf2/ARE, GCLM, HO-1, NQO1	[14,15]
Naringenin	Flavanone	NGNPs (25 mg/kg, i.v.), 2 h	12 h	ALT ≈ −98%, AST ≈ −87%	↓ AKP, liver volume, Bax, TUNEL^+^ cells ↑ Bcl-2; hepatic blood flow restored	[16]
Silymarin	Flavonolignan	Silymarin (100 mg/kg/day, p.o.), from 1 h	7 days	ALT −41.8%, AST −52.7%	↓ LDH, BUN, SCr, NO ↑ GSH	[17]
		Silymarin (200 mg/kg/day), delayed therapy from 24 h	3–7 days	Not calculable; APAP-only and NAC groups did not survive to biochemistry	↓ TNF-α, IL-1β, NF-κB, MDA, iNOS ↑ GSH, SOD, GCLC, Nrf2, HO-1, NQO1; survival improved vs. NAC	[18]
Baicalin	Flavone glycoside	Baicalin (30 mg/kg, i.p.) 30 min; baicalin (40 mg/kg, p.o.) 6–18 h	16 h; 24–72 h	ALT ≈ −85%; ALT ≈ −50%, AST ≈ −40%	↓ p-ERK, IL-6, TNF-α, MPO, IL-17 ↑ Nrf2; NLRP3/IL-18 axis modulated	[19,20]
Oroxyloside	Flavone glucuronide	Oroxyloside (100 mg/kg, i.g.), 1 h	2–24 h	ALT ≈ −73%, AST ≈ −49%	↓ CYP2E1, CYP1A2, IL-6, IL-1β, ROS-mediated mitochondrial dysfunction	[21]
Narirutin	Flavanone glycoside	Narirutin (50 mg/kg, i.p.), 1 h	6 h	ALT ≈ −55%, AST ≈ −57%	↑ TFEB nuclear translocation and autophagy via PPP3/calcineurin ↓ APAP–cysteine adducts; survival improved	[22]
Tiliroside	Flavonol glycoside	Tiliroside (20 mg/kg/day, i.g.), 3 h or 6 h (effect lost at 9 h)	24 h	ALT ≈ −71% (3 h), ≈ −52% (6 h); AST ≈ −64% (3 h), ≈ −45% (6 h)	KEAP1 inhibition → ↑ NRF2, HO-1 ↓ necrotic area, MDA; no effect in *Nrf2* hepatocyte knockout	[23]
Taxifolin/dihydroquercetin	Flavanonol	TAX@pro-glycymicelles (80 mg/kg, p.o.), 2 h and 4 h; dihydroquercetin (4–40 mg/kg/day, i.g.) from 1 h, twice daily	6 h; 48 h	TAX micelles: ALT ≈ −97%, AST ≈ −93%; dihydroquercetin: decreased ALT and AST	↑ oral bioavailability vs. unformulated taxifolin ↓ necrosis, HMGB1 signalling	[24,25]
Hesperidin	Flavanone glycoside	Hes-MMs (100 mg/kg), therapeutic arm after APAP	24 h	ALT ≈ −57%, AST ≈ −61%	↓ mtDNA release, cGAS–STING activation ↑ mitochondrial membrane potential, MnSOD, NQO1	[26]
Galangin	Flavonol	Galangin nanoparticles (10 or 20 mg/kg/day), for 10 days	10 days	ALT −41%, AST −39%	↓ MDA dose-dependently ↑ Nrf2, GCLC, GSH; hepatocyte damage reduced	[27]
Epigallocatechin-3-gallate	Flavan-3-ol	EGM NPs (0.5–2.0 mg/kg, i.v.), 2 h	24 h	ALT ≈ −82%, AST ≈ −80%	↓ ROS, inflammation, endoplasmic reticulum stress, apoptosis; necrotic area reduced	[28]
Phloretin, 4-hydroxychalcone and 4′-hydroxychalcone	Dihydrochalcone and chalcones	Phloretin, 4-HC or 4′-HC (25–100 mg/kg, i.p.), every 6 h after APAP	48 h	Decreased SGOT and SGPT	↓ mortality after a lethal APAP dose, from ≈75% to ≈20% at 25–50 mg/kg ↓ SGOT, SGPT	[29]

↑, increased; ↓, decreased, relative to the APAP-only group.

## Data Availability

No new data were created or analyzed in this study. Data sharing is not applicable to this article.

## Supplementary Materials

The following supporting information can be downloaded at: https://www.mdpi.com/article/10.3390/molecules31193527/s1, Table S1: Search strategies for PubMed, Scopus, Web of Science Core Collection and Embase, with the date of each search and the number of records retrieved; Table S2: Standardized data extraction form applied to the included studies; Table S3: Records identified per database and the number of duplicates removed.
