# Supplementary material for: Hepatoprotective Effects of Flavonoids Against Acetaminophen Toxicity
Source: Molecules. 2026 Oct 3;31(19):3527. doi: 10.3390/molecules31193527 (PMC13666650; doi:10.3390/molecules31193527)
Supplement: Supplementary file 1 [file molecules-31-03527-s001.zip › molecules-4559468-supplementary.pdf]

**Supplementary Information**

**Table S1. Search strategies for PubMed, Scopus, Web of Science Core Collection and Embase, with the date of each search and the number of records retrieved. (a) PubMed: Medical Subject Headings (MeSH) and keywords.**

Title: Hepatoprotective Effects of Flavonoids Against Acetaminophen Toxicity

Keyword:

Flavonoids: Flavonoid*

Mesh: "Flavonoids"[Mesh]

Total: (Flavonoid* OR "Flavonoids"[Mesh])

Acetaminophen: “N-Acetyl-p-aminopheno”l OR “Acetamidopheno”l OR “Acetominophen” OR “APAP” OR “p-Acetamidophenol” OR “p-Hydroxyacetanilide” OR “Paracetamol” OR “Hydroxyacetanilide” OR “N-(4-Hydroxyphenyl)acetanilide” OR “Algotropyl” OR “Acamol” OR “Anacin-3” OR “Anacin3” OR “Anacin 3” OR “Datril” OR “Panadol” OR “Tylenol” OR “Acetaco” OR “Acephen” OR

Mesh: ( "Acetaminophen/administration and dosage"[Mesh] OR "Acetaminophen/adverse effects"[Mesh] OR "Acetaminophen/agonists"[Mesh] OR "Acetaminophen/chemistry"[Mesh] OR "Acetaminophen/metabolism"[Mesh] OR "Acetaminophen/pharmacokinetics"[Mesh] OR "Acetaminophen/pharmacology"[Mesh] OR "Acetaminophen/poisoning"[Mesh] OR "Acetaminophen/therapeutic use"[Mesh] OR "Acetaminophen/toxicity"[Mesh] )

Total: (“N-Acetyl-p-aminophenol” OR “Acetamidophenol” OR “Acetominophen” OR “APAP” OR “p-Acetamidophenol” OR “p-Hydroxyacetanilide” OR “Paracetamol” OR “Hydroxyacetanilide” OR “N-(4-Hydroxyphenyl)acetanilide” OR “Algotropyl” OR “Acamol” OR “Anacin-3” OR “Anacin 3” OR “Datril” OR “Panadol” OR “Tylenol” OR “Acetaco” OR “Acephen” OR "Acetaminophen/administration and dosage"[Mesh] OR "Acetaminophen/adverse effects"[Mesh] OR "Acetaminophen/agonists"[Mesh] OR "Acetaminophen/chemistry"[Mesh] OR "Acetaminophen/metabolism"[Mesh] OR "Acetaminophen/pharmacokinetics"[Mesh] OR "Acetaminophen/pharmacology"[Mesh] OR "Acetaminophen/poisoning"[Mesh] OR "Acetaminophen/therapeutic use"[Mesh] OR "Acetaminophen/toxicity"[Mesh] )

Liver injury: “hepatotoxicity” OR “Liver poisoning” OR “Hepatic poisoning” OR “Hepatic Injury” OR “drug induced liver toxicity” OR “Liver Toxicity” OR “Drug induced liver poisoning” OR “Drug induced hepatictoxicity” OR

Mesh: ("Chemical and Drug Induced Liver Injury, Chronic"[Mesh] OR "Chemical and Drug Induced Liver Injury"[Mesh] OR "Chemical and Drug Induced Liver Injury/complications"[Mesh] OR "Chemical and Drug Induced Liver Injury/diagnosis"[Mesh] OR "Chemical and Drug Induced Liver Injury/drug therapy"[Mesh] OR "Chemical and Drug Induced Liver Injury/etiology"[Mesh] OR "Chemical and Drug Induced Liver

Injury/genetics"[Mesh] OR "Chemical and Drug Induced Liver Injury/history"[Mesh] OR "Chemical and Drug Induced Liver Injury/immunology"[Mesh] OR "Chemical and Drug Induced Liver Injury/metabolism"[Mesh] OR "Chemical and Drug Induced Liver Injury/mortality"[Mesh] OR "Chemical and Drug Induced Liver Injury/nursing"[Mesh] OR "Chemical and Drug Induced Liver Injury/pathology"[Mesh] OR "Chemical and Drug Induced Liver Injury/prevention and control"[Mesh] OR "Chemical and Drug Induced Liver Injury/surgery"[Mesh] OR "Chemical and Drug Induced Liver Injury/therapy"[Mesh] )

Total: ("hepatotoxicity" OR "Liver poisoning" OR "Hepatic poisoning" OR "Hepatic Injury" OR "drug induced liver toxicity" OR "Liver Toxicity" OR "Drug induced hepatotoxicity" OR "Chemical and Drug Induced Liver Injury, Chronic"[Mesh] OR "Chemical and Drug Induced Liver Injury"[Mesh] OR "Chemical and Drug Induced Liver Injury/complications"[Mesh] OR "Chemical and Drug Induced Liver Injury/diagnosis"[Mesh] OR "Chemical and Drug Induced Liver Injury/drug therapy"[Mesh] OR "Chemical and Drug Induced Liver Injury/etiology"[Mesh] OR "Chemical and Drug Induced Liver Injury/genetics"[Mesh] OR "Chemical and Drug Induced Liver Injury/history"[Mesh] OR "Chemical and Drug Induced Liver Injury/immunology"[Mesh] OR "Chemical and Drug Induced Liver Injury/metabolism"[Mesh] OR "Chemical and Drug Induced Liver Injury/mortality"[Mesh] OR "Chemical and Drug Induced Liver Injury/nursing"[Mesh] OR "Chemical and Drug Induced Liver Injury/pathology"[Mesh] OR "Chemical and Drug Induced Liver Injury/prevention and control"[Mesh] OR "Chemical and Drug Induced Liver Injury/surgery"[Mesh] OR "Chemical and Drug Induced Liver Injury/therapy"[Mesh] )

Final MeSH: **(((Flavonoid* OR "Flavonoids"[Mesh])) AND (("N-Acetyl-p-aminophenol" OR "Acetamidophenol" OR "Acetominophen" OR "APAP" OR "p-Acetamidophenol" OR "p-Hydroxyacetanilide" OR "Paracetamol" OR "Hydroxyacetanilide" OR "N-(4-Hydroxyphenyl)acetanilide" OR "Algotropyl" OR "Acamol" OR "Anacin-3" OR "Anacin 3" OR "Datril" OR "Panadol" OR "Tylenol" OR "Acetaco" OR "Acephen" OR "Acetaminophen/administration and dosage"[Mesh] OR "Acetaminophen/adverse effects"[Mesh] OR "Acetaminophen/agonists"[Mesh] OR "Acetaminophen/chemistry"[Mesh] OR "Acetaminophen/metabolism"[Mesh] OR "Acetaminophen/pharmacokinetics"[Mesh] OR "Acetaminophen/pharmacology"[Mesh] OR "Acetaminophen/poisoning"[Mesh] OR "Acetaminophen/therapeutic use"[Mesh] OR "Acetaminophen/toxicity"[Mesh] ))) AND (("hepatotoxicity" OR "Liver poisoning" OR "Hepatic poisoning" OR "Hepatic Injury" OR "drug induced liver toxicity" OR "Liver Toxicity" OR "Drug induced hepatotoxicity" OR "Chemical and Drug Induced Liver Injury, Chronic"[Mesh] OR "Chemical and Drug Induced Liver Injury"[Mesh] OR "Chemical and Drug Induced Liver Injury/complications"[Mesh] OR "Chemical and Drug Induced Liver Injury/diagnosis"[Mesh] OR "Chemical and Drug Induced Liver Injury/drug therapy"[Mesh] OR "Chemical and Drug Induced Liver Injury/etiology"[Mesh] OR "Chemical and Drug Induced Liver Injury/genetics"[Mesh] OR "Chemical and Drug Induced Liver Injury/history"[Mesh] OR "Chemical and Drug Induced Liver Injury/immunology"[Mesh] OR "Chemical and Drug Induced Liver Injury/metabolism"[Mesh] OR "Chemical and Drug Induced Liver**

**Injury/mortality"[Mesh] OR "Chemical and Drug Induced Liver Injury/nursing"[Mesh] OR "Chemical and Drug Induced Liver Injury/pathology"[Mesh] OR "Chemical and Drug Induced Liver Injury/prevention and control"[Mesh] OR "Chemical and Drug Induced Liver Injury/surgery"[Mesh] OR "Chemical and Drug Induced Liver Injury/therapy"[Mesh] ))** Filters: **from 2010 - 2025**

(("flavonoid*"[All Fields] OR "Flavonoids"[MeSH Terms]) AND ("N-Acetyl-p-aminophenol"[All Fields] OR "Acetamidophenol"[All Fields] OR "Acetominophen"[All Fields] OR "APAP"[All Fields] OR "p-Acetamidophenol"[All Fields] OR "p-Hydroxyacetanilide"[All Fields] OR "Paracetamol"[All Fields] OR "Hydroxyacetanilide"[All Fields] OR "n 4 hydroxyphenyl acetanilide"[All Fields] OR "Algotropyl"[All Fields] OR "Acamol"[All Fields] OR "anacin 3"[All Fields] OR "anacin 3"[All Fields] OR "Datril"[All Fields] OR "Panadol"[All Fields] OR "Tylenol"[All Fields] OR "Acetaco"[All Fields] OR "Acephen"[All Fields] OR "acetaminophen/administration and dosage"[MeSH Terms] OR "acetaminophen/adverse effects"[MeSH Terms] OR "acetaminophen/agonists"[MeSH Terms] OR "acetaminophen/chemistry"[MeSH Terms] OR "acetaminophen/metabolism"[MeSH Terms] OR "acetaminophen/pharmacokinetics"[MeSH Terms] OR "acetaminophen/pharmacology"[MeSH Terms] OR "acetaminophen/poisoning"[MeSH Terms] OR "acetaminophen/therapeutic use"[MeSH Terms] OR "acetaminophen/toxicity"[MeSH Terms]) AND ("hepatotoxicity"[All Fields] OR "Liver poisoning"[All Fields] OR "Hepatic poisoning"[All Fields] OR "Hepatic Injury"[All Fields] OR "drug induced liver toxicity"[All Fields] OR "Liver Toxicity"[All Fields] OR "Drug induced hepatotoxicity"[All Fields] OR "chemical and drug induced liver injury, chronic"[MeSH Terms] OR "Chemical and Drug Induced Liver Injury"[MeSH Terms] OR "chemical and drug induced liver injury/complications"[MeSH Terms] OR "chemical and drug induced liver injury/diagnosis"[MeSH Terms] OR "chemical and drug induced liver injury/drug therapy"[MeSH Terms] OR "chemical and drug induced liver injury/etiology"[MeSH Terms] OR "chemical and drug induced liver injury/genetics"[MeSH Terms] OR "chemical and drug induced liver injury/history"[MeSH Terms] OR "chemical and drug induced liver injury/immunology"[MeSH Terms] OR "chemical and drug induced liver injury/metabolism"[MeSH Terms] OR "chemical and drug induced liver injury/mortality"[MeSH Terms] OR "chemical and drug induced liver injury/nursing"[MeSH Terms] OR "chemical and drug induced liver injury/pathology"[MeSH Terms] OR "chemical and drug induced liver injury/prevention and control"[MeSH Terms] OR "chemical and drug induced liver injury/surgery"[MeSH Terms] OR "chemical and drug inuced liver injury/therapy"[MeSH Terms])) AND (2010:2025[pdat])

Papers identified: 220

**Table S1 (continued). (b) Scopus, Web of Science Core Collection and Embase.**

All searches were run on 18 September 2026 and limited to 2010–2025 and to publications in English. Medical Subject Headings have no equivalent in Scopus or Web of Science, so their entry terms were entered as free-text terms; Emtree terms were used in Embase.

### Scopus (Advanced search)

```
TITLE-ABS-KEY(flavonoid*) AND TITLE-ABS-KEY(acetaminophen OR paracetamol OR apap OR "n-acetyl-p-aminophenol" OR acetamidophenol OR "p-hydroxyacetanilide" OR hydroxyacetanilide OR tylenol OR panadol) AND TITLE-ABS-KEY(hepatotoxicity OR "liver toxicity" OR "hepatic injury" OR "liver injury" OR "liver poisoning" OR "hepatic poisoning" OR "drug induced liver injury" OR "drug-induced liver injury" OR "drug induced hepatotoxicity") AND PUBYEAR > 2009 AND PUBYEAR < 2026 AND LANGUAGE(english)
```

Records retrieved: 378

### Web of Science Core Collection (Advanced search)

```
TS=(flavonoid*) AND TS=(acetaminophen OR paracetamol OR APAP OR "N-acetyl-p-aminophenol" OR acetamidophenol OR "p-hydroxyacetanilide" OR hydroxyacetanilide OR tylenol OR panadol) AND TS=(hepatotoxicity OR "liver toxicity" OR "hepatic injury" OR "liver injury" OR "liver poisoning" OR "hepatic poisoning" OR "drug induced liver injury" OR "drug-induced liver injury" OR "drug induced hepatotoxicity") AND PY=(2010-2025) AND LA=(English)
```

Records retrieved: 230

### Embase (Ovid, 1974 to 16 September 2026)

```
1  flavonoid*.mp. or exp flavonoid/

2  (acetaminophen or paracetamol or APAP or n-acetyl-p-aminophenol or acetamidophenol or p-hydroxyacetanilide or hydroxyacetanilide or tylenol or panadol).mp. or exp paracetamol/

3  (hepatotoxicity or liver toxicity or hepatic injury or liver injury or liver poisoning or hepatic poisoning or drug induced liver injury or drug-induced liver injury or drug induced hepatotoxicity).mp. or exp liver toxicity/ or exp liver injury/

4  1 and 2 and 3

5  limit 4 to (english language and yr="2010 - 2025")

6  limit 5 to (article or article in press)
```

Records retrieved: 1099 at step 5; 924 at step 6

## Table S2. Standardized data extraction form applied to the included studies.

| | | | |
|---|---|---|---|
| Revision Date | | | |
| Reviewer ID | | | |
| Notes | | | |
| | **Eligible for Inclusion** (tick appropriate box) | | |
| | Yes | No | Unclear |
| **Type of Study**<br>Case-Control Study/Cohort Study/Observational Study | | | |
| **Type of Animal**<br>Mice | | | |
| **Type of Injury**<br>acetaminophen-induced liver injury (AILI) | | | |
| **Type of Intervention**<br>Post APAP- injury with flavonoids | | | |
| **Type of treatment**<br>Whole flavonoids | | | |
| Outcome Measures | | | |
| Sample Size | | | |
| Methods Summary | | | |
| Reported Outcomes | | | |
| Exclusion Reasons | | | |
| Comments | | | |

**Table S3. Records identified per database and the number of duplicates removed.**

| | |
|---|---|
| PubMed | 220 |
| Scopus | 378 |
| Web of Science | 230 |
| Embase | 924 |
| Total identified | 1752 |
| Duplicates removed | 668 |
| Unique records screened | 1084 |
